# Development and validation of a non-remission risk prediction model in First Episode Psychosis: An analysis of two longitudinal studies

**DOI:** 10.1192/bjo.2021.147

**Published:** 2021-06-18

**Authors:** Samuel Leighton, Pavan Mallikarjun, Rajeev Krishnadas, Jonathan Cavanagh, Simon Rogers, Rachel Upthegrove, Max Birchwood, Stephen Marwaha, Ewout Steyerberg, Georgios Gkoutos, Matthew Broome, Peter Liddle, Linda Everard, Swaran Singh, Nicholas Freemantle, David Fowler, Peter Jones, Vimal Sharma, Robin Murray, Til Wykes, Richard Drake, Iain Buchan, Shon Lewis

**Affiliations:** 1 University of Glasgow; 2 University of Birmingham; 3 NHS Greater Glasgow & Clyde; 4 University of Warwick; 5 University of Leiden; 6 University of Nottingham; 7 Neuropsychiatry - The Barberry Center; 8 University College London; 9 University of Sussex; 10 University of Cambridge; 11 University of Chester; 12 Institute of Psychiatry, Psychology and Neuroscience, King's College London; 13 University of Manchester; 14 University of Liverpool

## Abstract

**Aims:**

Psychosis is a major mental illness with first onset in young adults. The prognosis is poor in around half of the people affected, and difficult to predict. The few tools available to predict prognosis have major weaknesses which limit their use in clinical practice. We aimed to develop and validate a risk prediction model of symptom non-remission in first-episode psychosis.

**Method:**

Our development cohort consisted of 1027 patients with first-episode psychosis recruited between 2005 to 2010 from 14 early intervention services across the National Health Service in England. Our validation cohort consisted of 399 patients with first-episode psychosis recruited between 2006 to 2009 from a further 11 English early intervention services. The one-year non-remission rate was 52% and 54% in the development and validation cohorts, respectively. Multivariable logistic regression was used to develop a risk prediction model for non-remission, which was externally validated.

**Result:**

The prediction model showed good discrimination (C-statistic of 0.74 (0.72, 0.76) and adequate calibration with intercept alpha of 0.13 (0.03, 0.23) and slope beta of 0.99 (0.87, 1.12). Our model improved the net-benefit by 16% at a risk threshold of 50%, equivalent to 16 more detected non-remitted first-episode psychosis individuals per 100 without incorrectly classifying remitted cases.

**Conclusion:**

Once prospectively validated, our first episode psychosis prediction model could help identify patients at increased risk of non-remission at initial clinical contact.

